# Comparison of occlusion break responses and vacuum rise times of phacoemulsification systems

**DOI:** 10.1186/1471-2415-14-96

**Published:** 2014-07-30

**Authors:** Pooria Sharif-Kashani, Douglas Fanney, Val Injev

**Affiliations:** 1 Alcon Research, Ltd., 20511 Lake Forest Drive, Lake Forest, CA 92630, USA

**Keywords:** Phacoemulsification, Occlusion break surge, Vacuum rise time, Cataract, Compliance

## Abstract

**Background:**

Occlusion break surge during phacoemulsification cataract surgery can lead to potential surgical complications. The purpose of this study was to quantify occlusion break surge and vacuum rise time of current phacoemulsification systems used in cataract surgery.

**Methods:**

Occlusion break surge at vacuum pressures between 200 and 600 mmHg was assessed with the Infiniti® Vision System, the WhiteStar Signature® Phacoemulsification System, and the Centurion® Vision System using gravity-fed fluidics. Centurion Active Fluidics^TM^ were also tested at multiple intraoperative pressure target settings. Vacuum rise time was evaluated for Infiniti, WhiteStar Signature, Centurion, and Stellaris® Vision Enhancement systems. Rise time to vacuum limits of 400 and 600 mmHg was assessed at flow rates of 30 and 60 cc/minute. Occlusion break surge was analyzed by 2-way analysis of variance.

**Results:**

The Centurion system exhibited substantially less occlusion break surge than the other systems tested. Surge area with Centurion Active Fluidics was similar to gravity fluidics at an equivalent bottle height. At all Centurion Active Fluidics intraoperative pressure target settings tested, surge was smaller than with Infiniti and WhiteStar Signature. Infiniti had the fastest vacuum rise time and Stellaris had the slowest. No system tested reached the 600-mmHg vacuum limit.

**Conclusions:**

In this laboratory study, Centurion had the least occlusion break surge and similar vacuum rise times compared with the other systems tested. Reducing occlusion break surge may increase safety of phacoemulsification cataract surgery.

## Background

Cataract is a common vision-threatening condition that accounts for approximately 40% to 50% of global blindness; as such, an estimated 20 million cataract surgical procedures are performed worldwide each year [1-3]. Clinicians prefer ultrasound phacoemulsification of the crystalline lens because this approach has a low incidence of complications and better uncorrected visual outcomes than alternative treatment options for cataract removal [2,4-6]. Phacoemulsification is performed using a phacoemulsifier aspirator (PEA). Most PEA systems use peristaltic pump technology that allows for independent control of both the aspiration flow rate and vacuum limit and can facilitate markedly lower flow rates than those with nonperistaltic (ie, venturi) systems.

During normal surgical conditions (ie, when the phacoemulsification tip is not occluded), the vacuum level in the system’s fluidics is relatively low. When the tip becomes occluded with aspirated material during surgery, the vacuum in the system builds to a preset vacuum limit, which triggers a vacuum pump shut-off. When an occlusion break occurs, the vacuum accumulated in the aspiration lines returns to the original low level. In many systems, particularly older systems, a secondary effect referred to as occlusion break surge can occur.

**Figure 1:**
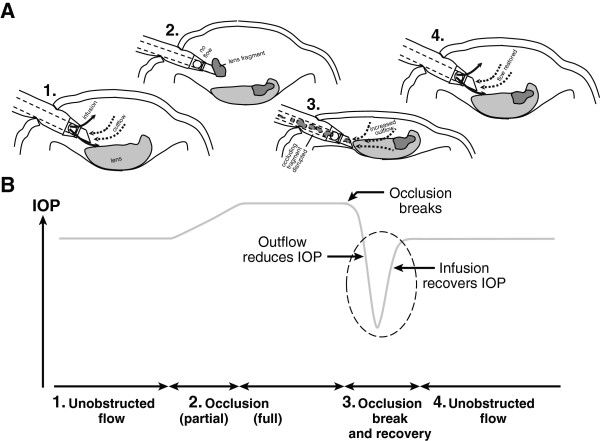
**Occlusion break surge: gravity-based systems.** The schematic depicts **(A)** the anterior chamber and **(B)** IOP. During unobstructed flow, aspiration and infusion are balanced to maintain a stable anterior chamber (A1) and IOP (B1). When the phaco tip becomes occluded with nuclear material, fluid flow is blocked (A2) and IOP increases (B2). With occlusion break, vacuum stored in aspiration tubing during occlusion (ie, no flow) can cause a sudden increase in aspiration rate (A3) and lead to a drop in IOP (B3). Infusion during unobstructed flow after occlusion break leads to recovery of the anterior chamber (A4) and IOP (B4). IOP = intraoperative pressure.

Occlusion break surge (Figure 1) is defined as when ocular fluid rushes into the aspiration port after the material occluding the phacoemulsification tip clears [7], filling the vacuum in the tubing and causing a subsequent drop in intraocular pressure [8]. Occlusion break surge can cause the anterior chamber to shallow [9,10] or cause the iris or posterior capsule to move toward the phacoemulsification tip, increasing the potential risk of posterior capsule rupture or iris trauma [5,11].

Ultrasound phacoemulsification technologies have evolved over the last several years to improve surgical efficacy and postsurgical outcome and to reduce complications of cataract surgery [12]. The Centurion® Vision System (Alcon Laboratories, Inc., Fort Worth, TX) is one of the newer PEA systems; this system utilizes smaller aspiration tubing and a cassette design with low compliance to minimize surge. Minimizing compliance (ie, increasing the rigidity) of the aspiration tubing can increase the rate of vacuum rise during occlusion (referred to as vacuum rise time), reaching the preset vacuum limit more quickly [10]. Low-compliance fluidic tubing also reduces occlusion break surge by decreasing the potential energy via vacuum that is stored in the tubing during occlusion [10]. The Centurion system also features Active Fluidics™ designed to provide greater stability of intraoperative pressure (IOP), minimizing IOP fluctuations that occur during occlusion onset and postocclusion break events.

The objective of this study was to measure, in a controlled laboratory setting, occlusion break surge and vacuum rise time of current phacoemulsification systems used in cataract surgery. This manuscript describes the testing and determination of occlusion break surge area and vacuum rise times of 4 PEA systems under surgically-relevant operating conditions.

## Methods

This laboratory study used no human or animal subjects and required no ethics approval.

### Equipment

Standard equipment, including a Foxboro pressure/vacuum transducer box (Invensys Systems, Inc., Plano, TX) and a digital storage oscilloscope (Classic 6000; Gould Instrument Systems, Cleveland, OH), was used for assessments of occlusion break surge and vacuum rise time. The infusion bottle height was set at the same actual elevation (90 cm) above the transducer for all systems tested to avoid potential differences in display height of the different systems. To eliminate variability due to differences in selected aspiration orifice cross-section areas, the same phacoemulsification handpiece (OZil®; Alcon Laboratories), phacoemulsification tip type (30^o^R, 0.9 mm, nonaspiration bypass system; Alcon Laboratories), and matching infusion sleeve type were used for all testing [13]. The fittings for irrigation and aspiration tubing on each PEA system used a standard Luer interface that enabled attachment of a single tip/handpiece configuration to all systems. This setup ensured that resistance, which is influenced by the area of the aspiration orifice, was identical among PEA systems. After each system was tuned and primed, the infusion fitting was tied to the infusion Luer connector on the OZil ultrasound handpiece. The aspiration fitting of the system was connected to the corresponding fitting located at the back of the handpiece.

Phacoemulsification systems and their respective fluidics and consumables are summarized in Table 1. Occlusion break surge was assessed using Centurion, Infiniti® (Alcon Laboratories), and WhiteStar® Signature (Abbott Medical Optics [AMO], Santa Ana, CA) phacoemulsification systems. Because flow rate cannot be directly controlled in a venturi pump system, occlusion break surge testing was performed using only peristaltic pump systems. Vacuum rise times were evaluated for Centurion, Infiniti, WhiteStar Signature, and Stellaris® Vision Enhancement System (Bausch & Lomb, Rochester, NY).

**Table 1:** Phacoemulsification systems tested

**Phacoemulsification system**	**Fluidics**	**Consumables**^†^	**Occlusion break**	**Vacuum rise**
Centurion	Active*; 40 (54), 50 (68), 55 (78), 65 (88.4)	Active Fluidics packs, P1423726	X	X^‡^
Centurion	Gravity; 90 cm H_2_O	Gravity packs, P1423727H	X	X
Infiniti	Gravity; 90 cm H_2_O	Intrepid Plus gravity packs	X	X
WhiteStar Signature	Gravity; 90 cm H_2_O	OPO70 tubing packs	X	X
Stellaris	Gravity; 90 cm H_2_O	BL5111 venturi packs	-	X

X = tested.

*Active Fluidics settings reflect target intraoperative pressure (IOP) and are presented as IOP setting, mmHg (equivalent bottle height, cm H_2_O).

^†^Occlusion break surge was assessed using 6 fluidics packs per system, including tubing and cassettes, tested in triplicate. Vacuum rise was assessed using a minimum of 3 fluidics packs per system.

^‡^IOP target of 65 mmHg only.

### Occlusion break surge testing

**Figure 2:**
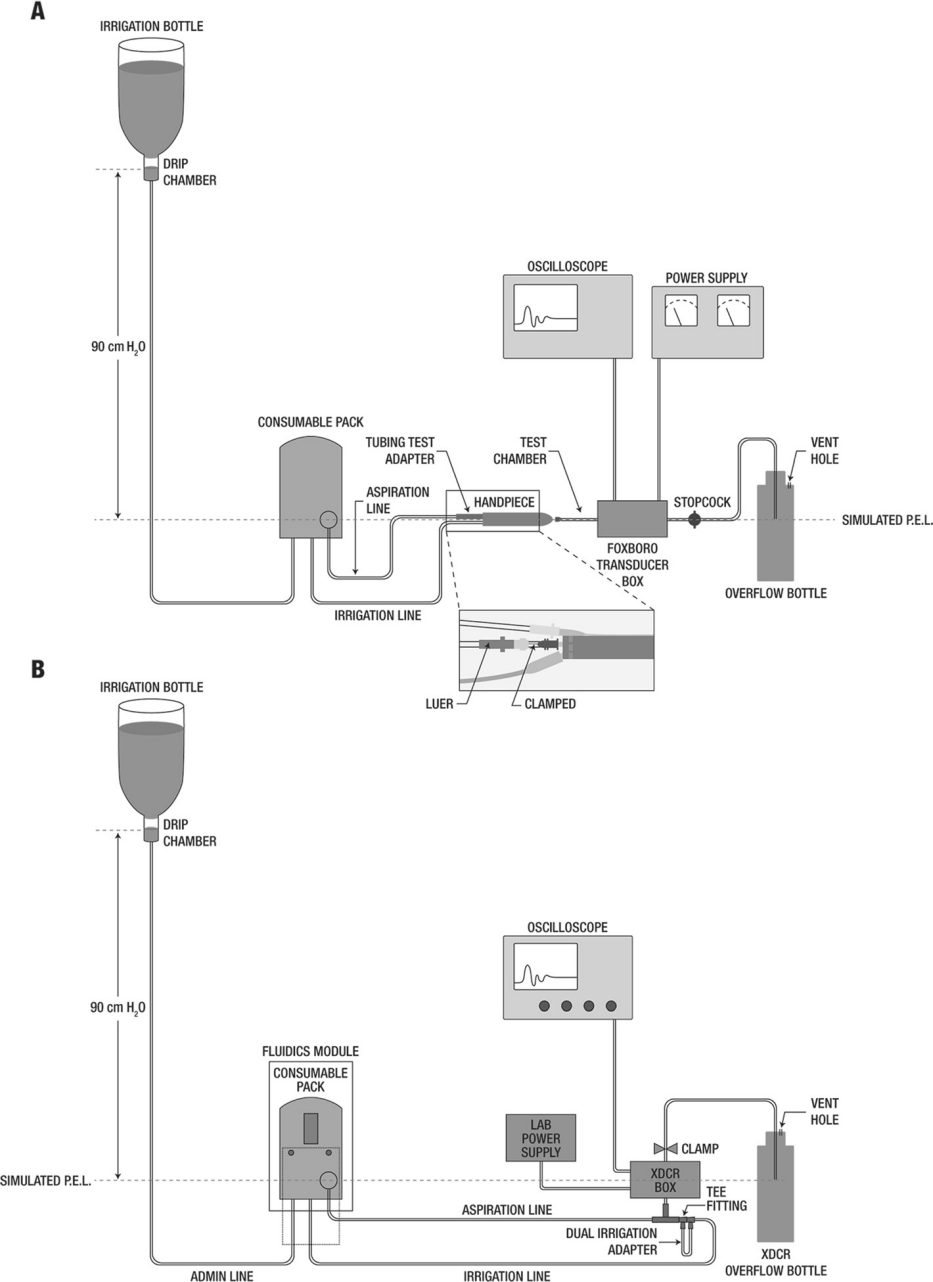
**Experimental setup for (A) occlusion break surge and (B) vacuum rise time testing.** Tubing was clamped at the sites indicated with arrows. PEL = patient eye level; XDCR = transducer.

The magnitude of the occlusion break surge was assessed using a published test protocol [13]. To ensure reproducibility of results, 6 fluidic packs (ie, cassettes and tubing) were tested per system, with each pack tested 3 times. Cassettes were primed to remove entrapped air, and the ultrasound tip and infusion sleeve were installed on the handpiece with the aspiration line attached. The oscilloscope was calibrated to the transducer; the vertical amplitude scale was set to 100 mV per division, and the horizontal time scale was set to 200 milliseconds per division. For testing systems with gravity fluidics, the irrigation bottle was placed at a height of 90 cm above the transducer midpoint. The Centurion system was also tested with Active Fluidics across target IOP settings ranging from 40 to 65 mmHg (equivalent to a bottle height of approximately 54–88 cm H_2_O; Table 1). Centurion system default parameters (vacuum rise = 0; IOP ramp = 1.0; irrigation factor = 1.0) were used for testing; modifications to these settings can be made directly from the front panel of the PEA system.All systems were tested at preset aspiration vacuum limits of 200, 300, 400, 500, and 600 mmHg, with an aspiration flow rate of 30 cc/minute for the peristaltic systems. The handpiece, transducer, and tubing were positioned at the system target patient eye level, and the test setup (Figure 2A), including tubing, test adapter, handpiece, test chamber assembly, and transducer box, was primed by activating the system’s foot pedal before each experiment. Aspiration flow was started by activating the foot pedal, and the tubing portion between the aspiration tubing test adapter and the handpiece was occluded using needle-nose pliers. Occlusion was released when the preset vacuum limit was reached. The simulated occlusion break surge event was captured with the oscilloscope; surge area, which accounts for both amplitude and duration of the surge, was determined from the area under the surge curve and above the datum line corresponding to positive chamber pressure of 20 mmHg.

### Vacuum rise time

Vacuum rise times were evaluated using a minimum of 3 packs per system, with packs primed on the respective phacoemulsification systems. The oscilloscope vertical amplitude scale was set to 500 mV per division, and the horizontal time scale was set to 1 second per division. The equipment setup for vacuum rise testing is depicted in Figure 2B. As described elsewhere [13], testing of phacoemulsification systems was performed at a bottle height of 90 cm for gravity-fed fluidics and target IOP of 65 mmHg for Active Fluidics (Centurion only; Table 1).

Vacuum rise times were evaluated at preset vacuum pressure limits of 400 and 600 mmHg for all systems. With the exception of Stellaris, which does not allow control of aspiration flow rate, all systems were tested at aspiration flow rates of 30 and 60 cc/minute. Occlusion was formed by clamping the irrigation line within 1 inch of the Luer connector, and aspiration flow was started with the foot pedal. Vacuum was allowed to build for approximately 8 seconds before the system was vented by releasing the foot pedal; vacuum pressure level was recorded at 0.5, 1, 1.5, 2, 3, 4, 5, and 6 seconds.

### Data analysis and statistics

Mean occlusion break surge area and standard deviation (SD) were calculated for each phacoemulsification system. Mean vacuum rise across time points was calculated for each system. Statistical analysis was performed by 2-way analysis of variance (ANOVA), with *P* values ≤0.05 considered statistically significant.

## Results

### Occlusion break

**Figure 3:**
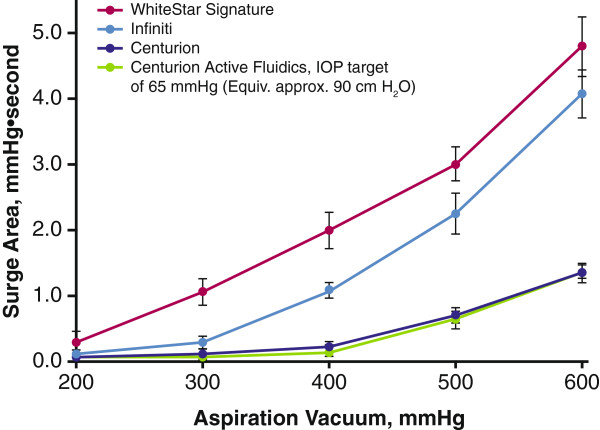
**Occlusion break surge responses with increasing aspiration vacuum.** Gravity fluidics (bottle height, 90 cm) was used unless otherwise indicated. IOP = intraoperative pressure.

Occlusion break surge increased with increasing aspiration vacuum for all phacoemulsification systems, with the smallest surge areas observed at 200 mmHg and largest surge areas observed at 600 mmHg. Across the aspiration vacuum pressure range tested, there was a significant difference in surge area between systems (2-way ANOVA, *P* < 0.05). Centurion produced substantially less occlusion break surge with gravity and Active Fluidics settings than the Infiniti and WhiteStar Signature; surge was highest with the WhiteStar Signature (Figure 3). At 400 mmHg, mean ± SD occlusion break surge areas were: WhiteStar Signature, 2.0 ± 0.28 mmHg∙second; Infiniti, 1.1 ± 0.12 mmHg∙second; Centurion (Active Fluidics, IOP target 65 mmHg), 0.2 ± 0.06 mmHg∙second; and Centurion (gravity fluidic), 0.1 ± 0.07 mmHg∙second. The difference in surge area produced by the 3 systems tested was largest at 600 mmHg. Surge areas were: WhiteStar Signature, 4.8 ± 0.45 mmHg∙second; Infiniti, 4.1 ± 0.37 mmHg∙second; Centurion Active Fluidics, 1.3 ± 0.15 mmHg∙second; and Centurion gravity fluidics, 1.4 ± 0.12 mmHg∙second. Centurion active (IOP target 65 mmHg) and gravity (90 cm H_2_O) fluidics performed similarly across the range of aspiration vacuum pressures tested.

**Figure 4:**
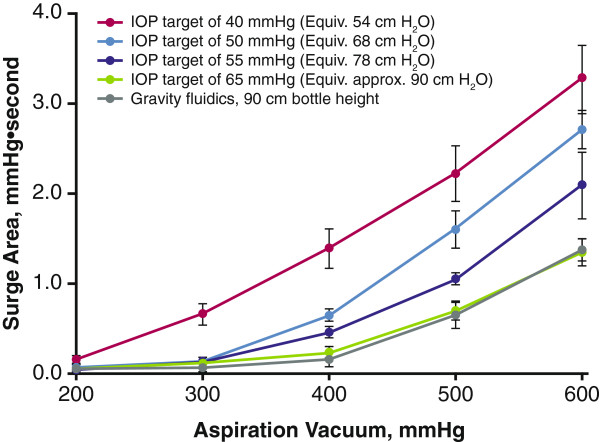
**Comparison of Centurion occlusion break surge response.** Gravity and Active Fluidics IOP target settings were used. IOP = intraoperative pressure.

At each preset aspiration vacuum pressure tested, occlusion break surge area predictably increased with decreasing Centurion Active Fluidics IOP target settings (Figure 4). This was most evident at higher aspiration vacuum pressures (ie, 400, 500, and 600 mmHg). Surge areas were similar with Active Fluidics target IOP settings of 50, 55, and 65 mmHg and gravity fluidics (90 cm H_2_O) at 200 and 300 mmHg (ranges, 0.04–0.06 and 0.05–0.13 mmHg, respectively). At 600 mmHg, all Centurion Active Fluidics target IOP settings achieved smaller occlusion break surge areas (Figure 4) than the gravity fluidics of Infiniti or WhiteStar Signature (Figure 3). With Active Fluidics, surge areas were 3.6 ± 0.37 mmHg with target IOP 40 mmHg; 2.7 ± 0.21 mmHg with target IOP 50 mmHg; 2.1 ± 0.37 mmHg with target IOP 55 mmHg; and 1.3 ± 0.15 mmHg with target IOP 65 mmHg.

### Vacuum rise time

**Figure 5:**
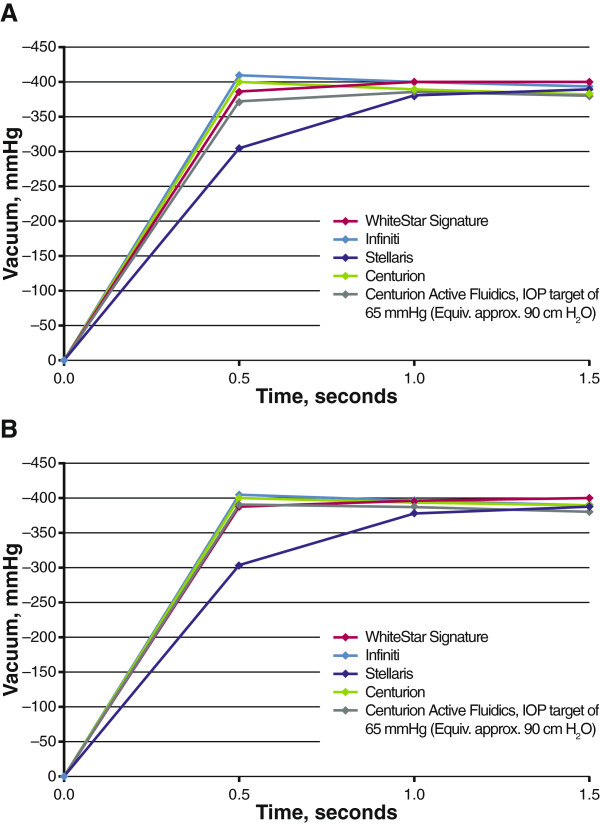
**Vacuum rise times at a vacuum limit of 400 mmHg.** Testing was performed with flow rates of **(A)** 30 cc/minute and **(B)** 60 cc/minute. Gravity fluidics (bottle height, 90 cm) was used unless otherwise indicated. IOP = intraoperative pressure.

**Figure 6:**
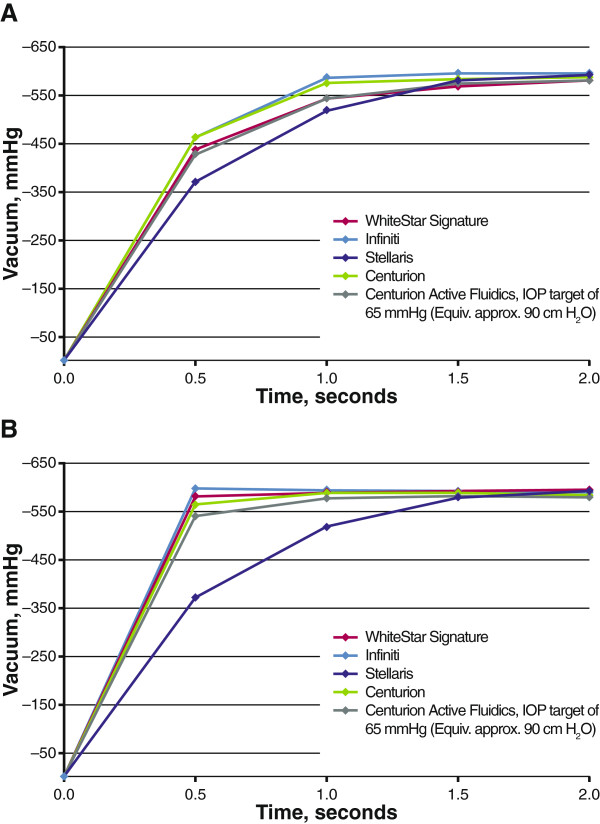
**Vacuum rise times at a vacuum limit of 600 mmHg.** Testing was performed with flow rates of **(A)** 30 cc/minute and **(B)** 60 cc/minute. Gravity fluidics (bottle height, 90 cm) was used unless otherwise indicated. IOP = intraoperative pressure.

With the vacuum limit preset to 400 mmHg, Infiniti had the fastest vacuum rise of the 4 systems tested during occlusion at flow rates of 30 and 60 cc/minute; the Stellaris, which does not enable flow rate control, had the slowest (Figure 5). Infiniti exceeded the preset vacuum limit at both tested flow rates (30 cc/minute, –410 mmHg; 60 cc/minute, –404 mmHg; both at 0.5 second). With gravity fluidics, Centurion demonstrated a rate of vacuum rise similar to that of Infiniti and did not exceed the preset vacuum limit (30 cc/minute, –399 mmHg; 60 cc/minute, –400 mmHg; 0.5 second each). At a flow rate of 60 cc/minute, vacuum rise times to 400 mmHg were similar between phacoemulsification systems with controllable flow rates.With the vacuum limit preset to 600 mmHg, Infiniti and WhiteStar Signature demonstrated the fastest initial vacuum rise during occlusion (Figure 6). No system reached the vacuum limit of 600 mmHg at 30 or 60 cc/minute. The maximum vacuum reached at a flow rate of 30 cc/minute was greatest with Infiniti (–595 mmHg, 1.5 seconds), followed by WhiteStar Signature (–593 mmHg, 5.0 seconds), Centurion with gravity fluidics (–587 mmHg, 2.0 seconds), and Centurion with Active Fluidics (–582 mmHg, 2 seconds). At 60 cc/minute, maximum vacuums achieved were –598 mmHg (Infiniti, 0.5 second), –595 mmHg (WhiteStar Signature, 1.5 seconds), –588 mmHg (Centurion with gravity fluidics, 1 second), and –583 mmHg (Centurion with Active Fluidics, 1.5 seconds). Stellaris reached –591 mmHg at 2 seconds.

## Discussion

Occlusion break surge occurs when potential energy via vacuum build-up in the aspiration line and cassette during phacoemulsification tip occlusion is suddenly released when the occlusion clears. In this study, the Centurion Vision System produced markedly less occlusion break surge than Infiniti or WhiteStar Signature, particularly at higher vacuum pressures. This was true whether gravity or comparable active Centurion fluidics were tested. Surge areas with Centurion Active Fluidics at a target IOP setting of 65 mmHg, which is equivalent to 88.4 cm H_2_O, and Centurion gravity fluidics at a bottle height of 90 cm H_2_O were similar at all aspiration vacuum pressures tested. Across the tested range of aspiration vacuum pressures, occlusion break surge with Centurion Active Fluidics settings equivalent to bottle heights of 54 to approximately 90 cm H_2_O was substantially smaller than with Infiniti or WhiteStar Signature gravity fluidics at 90 cm H_2_O. Vacuum rise times were generally similar for Infiniti, WhiteStar Signature, and Centurion at typical (30 cc/minute) and maximal (60 cc/minute) aspiration flow rates; vacuum rise with Stellaris, which does not have variable flow rate control, was slowest.

Phacoemulsification system compliance involves the system itself and its fluidic components, including aspiration tubing and cassette [10,13]. Reducing compliance of fluidics components is a key factor in managing occlusion break surge [8]. Occlusion break surge with Infiniti and WhiteStar Signature was comparable to that described previously [13]. WhiteStar Signature had a larger occlusion break surge than Infiniti, which is in agreement with the finding that Infiniti was the more compliant of the 2 systems in a laboratory setting [10]. In the typical working range of most cataract surgeries (ie, 200 − 600 mmHg) [13] and an irrigation bottle height of 90 cm H_2_O, surge areas with these systems were 2- to 7-fold and 3.5- to 20-fold higher, respectively, compared with surge area with the Centurion system. With traditional gravity fluidics, irrigation bottle height controls fluid infusion pressure [8]. With occlusion break, faster inflow is necessary to balance the ocular fluid outflow that fills the vacuum created in aspiration lines during occlusion, thereby reducing surge [14]. In this study, even at the highest preset aspiration vacuum level (600 mmHg) and lowest Active Fluidics irrigation fluid pressure (IOP target 40 mmHg, equivalent to 54 cm H_2_O), Centurion achieved decreased surge area compared with the gravity fluidics of Infiniti and WhiteStar Signature at 90 cm H_2_O. This was likely due to the lower-compliance fluidics of Centurion compared with the other systems tested. One effective approach to reduce occlusion break surge is to use a lower aspiration vacuum limit, thus lowering the maximum potential vacuum level in the aspiration fluidics.

Testing at vacuum limits of 400 and 600 mmHg and with typical (30 cc/minute) and maximal (60 cc/minute) flow rates provided information about the operational capabilities of the phacoemulsification systems investigated. Infiniti exceeded the 400-mmHg vacuum pressure limit, and no system achieved the 600-mmHg limit. With the exception of Stellaris, which had a slower rate of vacuum rise to both limits, vacuum rise times were similar among the systems and fluidics tested.

One strength of this study was that the previously published laboratory setup and use of the same phacoemulsification handpiece, tip, and sleeve for all systems tested enabled assessment of the relationship between each system’s fluidics and occlusion break surge without confounding variables [13]. Additionally, multiple fluidic cassettes were tested for each phacoemulsification system and demonstrated the reproducibility of system performance. The laboratory setup provided a standardized, repeatable test method using controlled, clinically relevant conditions. This study also included time as a factor in the occlusion break experiments, examining both the magnitude and duration of surge. Previous assessments have generally focused on surge magnitude alone [9,14,15].

## Conclusions

The Centurion system achieved less occlusion break surge when compared with Infiniti and WhiteStar Signature systems. Surge areas with the Centurion using gravity fluidics and a comparable Active Fluidics setting were similar at all aspiration vacuum levels tested. Vacuum rise times were similar for all the peristaltic systems tested; however, the Stellaris system demonstrated considerably slower vacuum rise than the other systems. Phacoemulsification systems with lower postocclusion surge may decrease the risk of complications during cataract surgery, whereas rapid vacuum limit response may increase efficiency.

## Abbreviations

ANOVA: Analysis of variance; IOP: Intraoperative pressure; PEA: Phacoemulsifier aspirator; SD: Standard deviation.

## Competing interests

PSK is a consultant to Alcon Research, Ltd., and DF and VI are employees of Alcon. DF and VI hold stock in Novartis, the parent company of Alcon. The authors have no other interests to disclose.

## Authors’ contributions

PSK, DF, and VI participated in the design and performance of the study and analysis of the data. All authors participated in drafting the manuscript, and read and approved the final version.

## Pre-publication history

The pre-publication history for this paper can be accessed here:

http://www.biomedcentral.com/1471-2415/14/96/prepub
